# Acceleration of callus formation during fracture healing using basic fibroblast growth factor-kidney disease domain-collagen-binding domain fusion protein combined with allogenic demineralized bone powder

**DOI:** 10.1186/s13018-015-0201-0

**Published:** 2015-05-09

**Authors:** Wataru Saito, Kentaro Uchida, Osamu Matsushita, Gen Inoue, Hiroyuki Sekiguchi, Jun Aikawa, Hisako Fujimaki, Masashi Takaso

**Affiliations:** Department of Orthopaedic Surgery, Kitasato University School of Medicine, 1-15-1 Minami-ku Kitasato, Sagamihara, Kanagawa Japan; Department of Bacteriology, Okayama University Graduate School of Medicine, Dentistry and Pharmaceutical Sciences, 2-5-1 Kita-ku Shikata-cho, Okayama, Japan

**Keywords:** Bone powder, Basic fibroblast growth factor, Collagen-binding domain, Fracture healing, Allogenic demineralized bone matrix

## Abstract

**Background:**

To repair fractures with large bone defects or gaps, demineralized allogenic bone matrix (DBM) is often applied to the fracture site. However, studies have shown that the use of DBM alone has limited efficacy for repairing fractures. In the present study, we developed an allogenic demineralized bone powder (DBP) with basic fibroblast-derived growth factor containing a polycystic kidney disease (PKD) domain and collagen-binding domain (CBD) from *Clostridium histolyticum* collagenase (ColH) and investigated the stimulatory effects of bFGF-PKD-CBD combined with allogenic DBP on bone growth in a mouse femur fracture model.

**Methods:**

DBP mixed with either phosphate-buffered saline (PBS) (DBP/PBS), 0.58 nmol basic fibroblast growth factor (bFGF) (0.58 nmol DBP/bFGF), 0.058 nmol bFGF-PKD-CBD (0.058 nmol DBP/bFGF-PKD-CBD), or 0.58 nmol bFGF-PKD-CBD (0.58 nmol DBP/bFGF-PKD-CBD) was grafted into fracture sites.

**Results:**

bFGF-PKD-CBD/DBP composite accelerates callus formation in a bone fracture model in mice and clearly showed that the composite also increases bone mineral density at fracture sites compared to bFGF/DBP. In addition, bFGF-PKD-CBD/DBP increased callus volume and bone mineral content to similar levels in fractures treated with a tenfold higher amount of bFGF at 4 weeks.

**Conclusions:**

Our results suggest that bFGF-PKD-CBD/DBP may be useful for promoting fracture healing in the clinical setting.

## Background

Certain types of fractures are associated with delayed union or nonunion and therefore require bone grafting [1-3]. Allogenic demineralized bone matrix (DBM) is a useful bone-filling material because it serves as biologic osteoconductive scaffold that retains the trabecular collagenous structure of the original tissue, and has bone morphogenetic protein 2 (BMP-2) retention/liberation properties [4,5]. Due to these properties, DBM is one of the most extensively used bone scaffolds in the clinical setting worldwide [1,6]. However, studies have shown that the use of DBM alone has limited efficacy for repairing bone defects [7,8].

To overcome this limitation, strategies for the repair of large fractures with DBM should include bone-inducing growth factors. A number of growth factors, such as BMP-2, basic fibroblast growth factor (bFGF), and osteogenic protein-1 (OP-1), have been studied for their potential to promote bone growth and union [9-14]. For example, BMP-2 and bFGF have been shown to accelerate the healing of fractures and bone defects [11,12], and OP-1 has been successfully used to treat tibial nonunions [9,10]. However, in practice, bone-inducing factors rapidly diffuse in body fluid and fall below therapeutic concentrations at defect sites. For this reason, large doses and/or repeated administrations of growth factors are required for sustained therapeutic effect, but such dosing regimens may be clinically impractical and expensive and can lead to adverse side effects [15,16]. To increase the osteogenic potential of bone-inducing growth factors, they should ideally be combined with a carrier, such as collagen or DBM, to promote their retention at fracture sites.

We previously fused the collagen-binding domain (CBD) and polycystic kidney disease (PKD) domain of *Clostridium histolyticum* class II collagenase (ColH) to bFGF and showed that the subcutaneous injection of this collagen-binding bFGF fusion protein (bFGF-PKD-CBD) without carrier into nude mice had more potent skin fibroblast growth-promoting effects at the injection site than native bFGF [17]. bFGF-PKD-CBD also markedly enhanced bone formation when loaded onto autologous DBM that was grafted onto intact rat femurs [18]. Based on these findings, we speculated that the combination of bFGF-PKD-CBD and DBM may promote the retention of bFGF at injury sites and thereby accelerate bone repair. However, the efficacy of this treatment approach has only been evaluated with autologous DBM and healthy bone, and the bone formation-promoting effects of bFGF-PKD-CBD in combination with allogenic DBM in bone injury models have not been determined.

Here, we investigated the stimulatory effects of bFGF-PKD-CBD combined with allogenic demineralized bone powder (DBP) on bone growth in a mouse femur fracture model.

## Methods

### Preparation of allogenic dematerialized bone powder

Both femurs were harvested from 36 C3H/HeN (H-2k) mice, and bone lipids were removed by treatment with chloroform/methanol. The harvested femoral bones were broken into small fragments, which were then passed through a 1-mm filter to collect the bone powder. To prepare DBP, the bone powder was demineralized using 0.6 N HCl for 18 h at 4°C. The particle size distributions were determined by laser scattering using a LMS-30 Micron Sizer (Seishin Enterprise Co., Ltd., Tokyo, Japan) and cumulative size distribution limits of D10, D50, and D90, which correspond to the percentage of particles (10%, 50%, and 90%, respectively) in a sample that is below a certain size. The surfaces of the DBP were observed by scanning transmission electron microscopy (SEM; JSM-7400F; JEOL Ltd., Tokyo, Japan).

### Preparation of bFGF and bFGF-PKD-CBD

Recombinant human bFGF was purchased from Kaken Pharmaceuticals (Tokyo, Japan). The construction of the fusion protein of bFGF, PKD, and the CBD derived from *C. histolyticum* class II collagenase (ColH) was previously described [18]. The biological activities of purified bFGF-PKD-CBD were confirmed using a proliferation assay with cultured periosteal mesenchymal cells [19]. bFGF-PKD-CBD exhibited the same cell proliferation ability as bFGF *in vitro*. The affinity of bFGF-PKD-CBD for allogenic DBP was confirmed *in vitro* by ELISA, as previously described [18]. In the assay, 0.064 nmol of bFGF-PKD-CBD bound to 1 mg of allogenic DBP.

### Fracture generation

All procedures involving the handling of animals adhered to the guidelines of the animal ethics committee of Kitasato University. A specific pathogen-free colony of C57BL/6J mice was housed in a semi-barrier system under controlled conditions (temperature, 23°C ± 2°C; humidity, 55% ± 10%; and lighting, 12-h light/dark cycle) throughout the study at Nippon Charles River Laboratories (Kanagawa, Japan). Mice were allowed access to standard rodent chow (CRF-1; Oriental Yeast Co., Ltd., Tokyo, Japan) and water *ad libitum*. Fractures were generated in the femurs of 72 C57BL/6J mice by first making a 4-mm medial parapatellar incision in the left knee under sterile conditions to laterally dislocate the patella [20]. A 0.5-mm hole was then drilled into the intracondylar notch, into which a nail (0.5 mm in diameter) was inserted retrograde for the creation of fractures, which were generated with a wire saw (0.22 mm in diameter) using a small lateral approach. After performing the fracture generation procedure, the nail was implanted deeply to stabilize the fracture. Soft x-rays were used to confirm the successful creation of fractures [20]. As we previously demonstrated that the combination of 0.58 nmol bFGF-PKD-CBD and collagen powder accelerated bone formation in mice [19], in the present study, the C57BL/6J mice were divided into four groups (18 mice/group), 8 mg DBP mixed with either phosphate-buffered saline (PBS) (DBP/PBS, control group), 0.58 nmol bFGF (0.58 nmol DBP/bFGF group), 0.058 nmol bFGF-PKD-CBD (0.058 nmol DBP/bFGF-PKD-CBD group), or 0.58 nmol bFGF-PKD-CBD (0.58 nmol DBP/bFGF-PKD-CBD group) was grafted into fracture sites. The mice were allowed to use their fractured leg without restriction immediately after the grafting procedure until micro-CT analysis was performed at 2, 4, and 6 weeks (*n* = 6, each time point).

### Quantification of the mineral content and volume of newly formed callus

Femurs and the surrounding muscle were excised from sacrificed mice at 2, 4, and 6 weeks after fracture generation and treatment and were then stored in 4% paraformaldehyde for 48 h at 4°C. Micro-CT images of whole femurs in PBS were obtained using a microfocus X-ray CT system (inspeXio SMX-90CT; Shimadzu Co., Ltd., Tokyo, Japan) and the following settings: acceleration voltage, 90 kV; current, 110 mA; voxel size, 20 μm/pixel; and matrix size, 1,024 × 1,024. From the obtained images, 10-mm regions of interest (500 slices) in the midfemur were defined. Three-dimensional (3D) image analysis software (Tri-3D-Bon; Ratoc System Engineering Co., Ltd, Tokyo, Japan) was used to measure new bone volume and bone mineral content in the defined regions, as previously described [21]. For assessing new bone volume and bone mineral content, a hydroxyapatite (HA) calibration curve was generated from the data obtained from phantom images prepared with 200, 300, 400, 500, 600, 700, and 800 mg HA/cm^3^. The bone mineral content in each sample was determined by comparing the measured densities in the micro-CT images to the HA calibration curve, and new bone was defined using a threshold value of ≥300 mg/cm^3^.

### Statistical analysis

Differences among the PBS, bFGF, and bFGF-PKD-CBD treatment groups were examined using one-way ANOVA with the Fisher’s least significant difference test. All statistical analyses were performed using SPSS software (Version 11.0; SPSS, Inc., Chicago, IL, USA). A *P* value of <0.05 was considered statistically significant.

## Results

### Characterization of allogenic DBP

**Figure 1:**
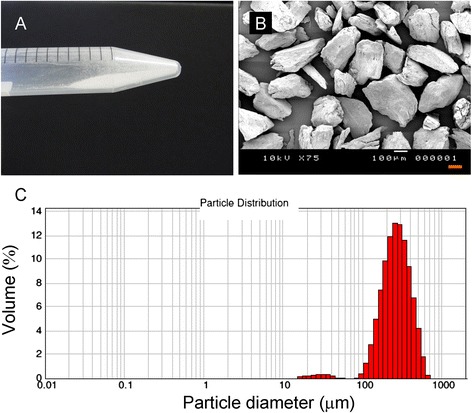
Appearance and SEM images of prepared demineralized allogenic bone powder. **(A)** Photograph of the prepared bone powder in a 1.5-cm diameter tube. **(B)** SEM image of the prepared bone powder. Scale bar indicates 100 μm. **(C)** Graph showing the size distribution of the prepared demineralized allogenic bone powder particles.

The prepared allogenic DBP was morphologically characterized by SEM, which showed that the particles appeared irregular in shape and had a rough surface (Figure 1A, B). Based on measurements of the diameter of the bone particles, the values for D10, D50, and D90 were determined to be 154.32, 266.11, and 439.94 μm, respectively (Figure 1C).

### Effect of bFGF-PKD-CBD-loaded DBP on callus formation

**Figure 2:**
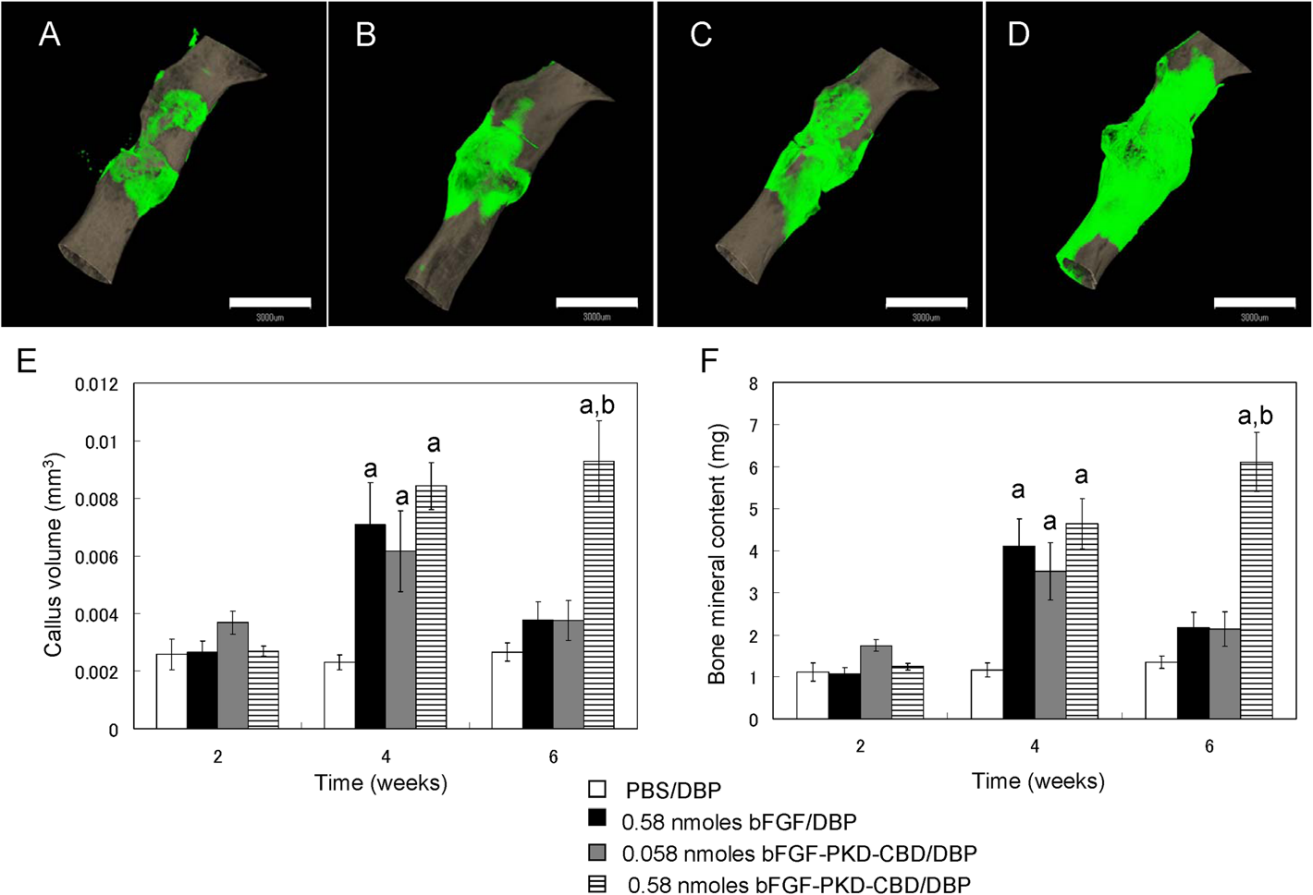
3D micro-CT analysis of femurs after grafting of demineralized allogenic bone powder loaded with bFGF-PKD-CBD. 3D micro-CT images of fractured mouse femurs treated with **(A)** PBS/DBP, **(B)** 0.58 nmol bFGF/DBP, **(C)** 0.058 nmol bFGF-PKD-CBD/DBP, and **(D)** 0.58 nmol bFGF-PKD-CBD/DBP at 6 weeks. Green: newly formed bone; gray: existing bone. The scale bars indicate 3 mm. **(E)** Callus area and **(F)** bone mineral content at fracture sites for the indicated treatment groups. Data are presented as the mean ± standard error (S.E.; error bars; *n* = 6). a: *P* < 0.05 compared with the control group (PBS/DBP). b: *P* < 0.05 compared with the 0.58 nmol bFGF group.

Callus formation at fracture sites in mice femurs grafted with DBP in PBS (PBS/DBP) or DBP loaded with bFGF (bFGF/DBP) or bFGF-PKD-CBD (0.058 nmol and 0.58 nmol bFGF-PKD-CBD/DBP) was evaluated after 2, 4, and 6 weeks by micro-CT imaging (Figure 2A–F). Similar amounts of callus formation and bone mineral content were observed for femurs treated with PBS/DBP, bFGF/DBP, 0.058 nmol bFGF-PKD-CBD/DBP, and 0.58 nmol bFGF-PKD-CBD/DBP at 2 weeks (Figure 2E, F). However, after 4 weeks, fracture sites grafted with 0.58 nmol bFGF/DBP and 0.058 or 0.58 nmol bFGF-PKD-CBD/DBP had significantly higher levels of callus volume and bone mineral content compared to those grafted with PBS/DBP (Figure 2E, F). After 6 weeks, the callus volume and bone mineral content in fracture sites grafted with 0.58 nmol bFGF-PKD-CBD/DBP were significantly higher than those found in the other three groups of treated femurs. In addition, no differences in callus formation or bone mineral content in fracture sites treated with PBS/DBP, bFGF/DBP, or 0.058 nmol bFGF-PKD-CBD/DBP femur were observed at 6 weeks (Figure 2E, F).

## Discussion

In mice, femur fracture sites grafted with 0.58 nmol bFGF-PKD-CBD/DBP had significantly higher levels of callus volume and bone mineral content at 4 weeks compared to fractures treated with DBP alone. In addition, treatment with a lower concentration of bFGF-PKD-CBD/DBP than that used for bFGF/DBP led to higher comparative callus volume and bone mineral content compared to fractures treated with DBP alone at 4 weeks. Notably, the callus volume and bone mineral content in fracture sites grafted with 0.58 nmol bFGF-PKD-CBD/DBP at 6 weeks were higher than those of the other three treatment groups. Taken together, these results suggest that bFGF-PKD-CBD/DBP may be useful for promoting fracture healing in the clinical setting.

Demineralized bone material is a useful bone-filling material due to its excellent BMP-2 retention/liberation properties, which promotes cell-mediated mineralized matrix deposition [22,23]. However, implanting DBM alone for the reconstruction of bone defects often fails to achieve repair [7]. To overcome this limitation, Aspenberg et al. [24] mixed allograft bone with bFGF and showed that this combination increased the calcium content of bone after implantation into defect sites in rats. Lu and Rabie [25] also demonstrated that the combination of allograft bone and bFGF accelerates bone formation in rabbit. We previously established a method for accelerating periosteal bone formation using autologous DBP decorated with collagen-binding bFGF protein fused with a PKD domain and CBD derived from *C. histolyticum* class II collagenase (ColH). In the present study, we demonstrated that the bFGF-PKD-CBD/DBP composite accelerates callus formation in a bone fracture model in mice and clearly showed that the composite also significantly increases the bone mineral density at fracture sites compared to bFGF/DBP. Notably, the callus volume and bone mineral content of fracture sites in the 0.58 nmol bFGF/DBP and 0.058 nmol bFGF-PKD-CBD/DBP treatment groups had decreased at 6 weeks compared to 4 weeks. This decrease was attributable to bone remodeling, which occurred when bone formation reached a plateau during the fracture healing process at approximately 4 weeks in these two treatment groups. In contrast, the callus volume and bone mineral content of femurs in the 0.58 nmol bFGF-PKD-CBD/DBP group increased throughout the 6-week recovery period, suggesting that the bFGF-PKD-CBD/DBP composite continuously stimulated bone formation through its increased retention at fracture sites due to the presence of the PKD and CBD.

In addition, bFGF-PKD-CBD/DBP increased callus volume and bone mineral content to similar levels in fractures treated with a tenfold higher amount of bFGF at 4 weeks. Taken together, these findings suggest that bFGF-PKD-CBD/DBP composite has greater fracture healing effects than bFGF alone, even at markedly lower concentrations. This property may therefore reduce the adverse effects of bFGF, such as thrombocytopenia, renal toxicity, and malignant cell activation [15,16], when used in the clinical setting.

During fracture healing, periosteal mesenchymal cells proliferate in the early phase and then differentiate into osteoblasts, which undergo expansion in the late phases and are involved in the synthesis of new bone. In the osteoinductive process, bFGF and BMP-2 play important roles in periosteal proliferation and osteogenic differentiation, respectively [26]. The osteogenic-promoting effects of synthetic bFGF and BMP-2 have also been reported [27-29]. For example, Akita et al. [27] showed that coadministration of BMP-2 and bFGF accelerates cranial bone defect healing. The combined delivery of BMP-2 and bFGF in the form of nanostructured colloidal gelatin gel also promotes bone regeneration in a rat femoral condyle defect model [28]. These findings suggest that the allogenic DBM/bFGF-PKD-CBD composite may stimulate both periosteal mesenchymal cell proliferation in the early phase of fracture healing due to the bFGF component and osteogenic differentiation in late phases due to the BMP-2 properties of DBM, leading to efficient fracture repair. Thus, allogenic DBM/bFGF-PKD-CBD is a promising composite material for accelerating bone formation during fracture healing in the clinical setting.

## Conclusions

We revealed that the anchoring of bFGF to demineralize allogenic DBP using the PKD and CBD derived from *C. histolyticum* collagenase effectively promotes fracture healing when this composite is applied to fracture sites. Specifically, the combination of DBP and the bFGF-PKD-CBD fusion protein increased callus volume and bone mineral content when grafted onto fractured mice femurs. bFGF-PKD-CBD/DBP composite is therefore a promising agent for promoting fracture healing in the clinical setting.
